# Coffee is protective against oral and pharyngeal cancer: A systematic review and meta-analysis

**DOI:** 10.4317/medoral.21829

**Published:** 2017-08-16

**Authors:** João Miranda, Luis Monteiro, Rui Albuquerque, José-Júlio Pacheco, Zahid Khan, Jose Lopez-Lopez, Saman Warnakulasuryia

**Affiliations:** 1 Medicine and Oral Surgery Department and Institute of Research and Advanced Training in Health Sciences and Technologies (IINFACTS), University Institute of Health Sciences (IUCS-N), CESPU, 4585-116 Paredes, Portugal; 2 Oral Medicine Department, Birmingham Dental Hospital & School of Dentistry, University of Birmingham. Mill Pool Way, B5 7EG. United Kingdom; 3 School of Dentistry, University of Barcelona, Oral Health and Masticatory System Group (Bellvitge Biomedical Research Institute) IDIBELL, L’Hospitalet de Llobregat, Barcelona 08907, Spain; 4 Oral Medicine, King’s College London, the WHO Collaborating Centre for Oral Cancer, London, United Kingdom

## Abstract

**Objectives:**

Certain Coffee is one of the most popular and consumable drinks worldwide. However, there are conflicting results on the influence of this drink in oral and pharyngeal cancer risk. To clarify this, we aimed to systemically review and carry out a meta-analysis of the relevant literature on the association between coffee and oral and pharyngeal cancer.

**Study Design:**

We carried out an electronic search of publications up to August 2016 from PubMed, National Library of Medicines Medline, Embase, Science Direct and the Cochrane Central Register. The Newcastle–Ottawa scale was used to address the quality of the studies a meta-analysis was carried out using random-effects models.

**Results:**

From the 22,515 entries identified in the search, 13 case-control and 4 cohort studies were selected. With regards to quality on the Newcastle-Ottawa scale, an overall value of 6.06 was obtained. The analysis for oral and pharyngeal cancer grouped together indicated a pooled OR of .69 (95% CI of .57-.84; *p*<001) for high versus low coffee consumption with a moderate heterogeneity (I2: 50.3%; *p*=.009). Regarding studies on oral cavity cancers we observed a pooled OR of 0.82; 95% CI =.58-1.16; *p*=.257) and for pharyngeal cancers a pooled OR of .72 (95% CI of 0.54-.95; *p*=.019). There was no significant publication bias.

**Conclusions:**

The results show an inverse association between high coffee consumption and the risk of oral and pharyngeal cancers, which indicates that coffee may have a protective role against these cancers. Further larger prospective observational cohort studies are needed to address any effect of other possible co-factors.

** Key words:**Coffee, caffeine, oral cancer, pharyngeal cancer, risk, meta-analysis.

## Introduction

Oral and pharyngeal cancer are the sixth most common cancers worldwide with a high mortality rate and overall poor for the survivors (1,2). The aetiology is multi-factorial, which includes tobacco use (smoked or chewed), alcohol consumption, human papillomavirus (HPV) and dietary factors (1,3). Multiple studies have been carried out to develop a diet which may have a posi-tive preventive effect on oral cancer (4).

Coffee, after water, is the leading beverage in the world which highlights the importance of knowledge of its possible influence on human health (5). The composition of coffee, besides caffeine, includes several antioxidants, polyphenols, cafestol, kahweol, chlorogenic acid and hydroxyhydroquinone (6). Some studies have suggested that these constituents could provide some genotoxicity protection thus classifying coffee as an anti-cancer agent (6).

The International Agency for Research On Cancer (IARC) in 1991 had previously classified coffee as possibly carcinogenic to humans (Group 2B). The large body of evidence currently available led the IARC to recently re-evaluate the carcinogenicity of coffee drinking. They concluded that there was inadequate evidence for the carcinogenicity of coffee drinking but did not refer to any protective effects of coffee consumption on oral or pharyngeal cancers (7).

The common use worldwide of this beverage led to several studies that tried to analyse the contribution of coffee consumption on the risk of oral cancer, although with conflicting results. Some studies reported an association of high coffee consumption to an augmented risk of oral cancer (8,9) and others showed an clear inverse association with the risk of oral cancer (10-14). Recently, there have been reports of a protective effect of coffee consumption on oral cancer from two recent meta-analysis (15,16). However, the primary studies included cases of pharyngeal cancers under the category of oral cancer, with no clear separation of both locations and therefore no comparison of the effects against the oral cavity versus the pharynx can be made (15,16). Consequently, it raised the study question: Does coffee have a protective effect in the etiology of oral cancer and pharyngeal cancers as a group and also as separate entities?

To answer this question, we aim to assess the impact of coffee drinking on oral cavity and pharyngeal cancer, by reviewing the relevant literature and carrying out a meta-analysis.

## Material and Methods

A systematic review and meta-analysis was carried out to retrieve and summarise the published literature on coffee consumption on the development of oral and pharyngeal cancer. An initial electronic search of the databases was conducted up to August of 2016, within PubMed, Embase, Science Direct and the Cochrane Central Register. We used a combination of keywords and control terms (MeSH) were used wherever possible. The search terms included “coffee” OR “caffeine” OR “beverage” OR “drink” OR “lifestyle” AND “mouth cancer” OR “oral cancer” OR “pharyngeal cancer” OR “pharynx cancer” OR “oropharynx cancer” OR “nasopharynx cancer” OR “hypopharynx cancer” OR “oropharyngeal cancer” OR “nasopharyngeal cancer” OR “hypopharyngeal cancer” AND “risk”. Additionally, other relevant sources were screened, including the reference list from the pool of included studies and on relevant review papers on the topic.

The inclusion criteria included observational studies (case-control or cohort studies, retrospective and prospective) in English, Portuguese, French and Spanish languages with regards to squamous cell carcinoma involving the oral cavity or pharynx and the association with coffee consumption. In order to eliminate the language bias, if an abstract or article was written in a different language, a request for translation of the article into English was carried out before allowing for a further assessment. Information on odds ratio (OR) or relative risk (RR) and respective 95% confidence interval (CI) or information to calculate them were considered mandatory. All articles which did not fit into the criteria were excluded, including reviews, author debates, letters to the editor or studies in which it was not clear of the impact of coffee consumption in the development of oral or pharyngeal cancer.

The title and abstracts from each article were identified via the search engines and were independently reviewed by the authors (JM, LM, RA, JP, ZK, JLL & SW) for consideration of inclusion within the systematic review. If insufficient information was provided in the abstract or if there was any doubt to include an article, then the full text was reviewed before reaching a consensus.

- Data Extraction

Individual measures were extracted from each selected study, using a standardised form. For each article, the following informa-tion was extracted: the principal author’s last name, year of publication, experimental design, country, sample size, cancer type, OR´s or RR´s with respective 95% CI´s regarding coffee consumption (highest vs lowest consumption) and covariates. Three main areas of analysis were performed: the first with the goal of estimating the overall effect of coffee consumption on oral cavity and pharyngeal cancers together, the second focused on the relationship between coffee consumption and oral cavity cancer (including articles with exclusive information on oral cavity cancers), and the last on the association between coffee consumption and pharyngeal cancer (including articles with exclusive information on pharyngeal cancers).

- Quality assessment

We evaluated the methodological quality of the selected studies by using the Newcastle–Ottawa scale (NOS) for observational studies. The check-list contained 9 items (regarding patient population and selection, comparability of the study, and exposure) with every item accounting for 1 point. Articles were independently assessed by authors (JM, LM, RA, JP, ZK, JLL & SW) and in case of disagreement, a final consensus would be reached. Supported by previous studies (17), articles with a final score of 5 or more points were included.

- Statistical analysis

The meta-analysis procedures were carried out by an experienced independent statistician. For each study, the meta-analytic procedures were implemented with Stata® software (version 12.0; Stata Corporation, College Station, TX). The Odds-Ratios (ORs) and the corresponding 95% CI were extracted from individual studies. For studies in which the effects were presented separately for different subgroups (e.g. male and female subjects), weighted averages of estimates were calculated. The presence of significant heterogeneity was statistically tested with the Cochran Q-test and I2 statistics. I2 values of 25, 50 and 75 represent low, moderate and high heterogeneity, respectively. In the presence of heterogeneity, subgroup analyses were conducted to explore whether the study design, country and number of cases had an impact in heterogeneity levels.

For each individual study, the OR for the highest versus the lowest level of coffee consumption were extracted. Using this approach, an OR value of 1 indicates an equal risk of oral and pharyngeal cancer between highest and lowest categories of coffee consumption; an OR greater than 1 demonstrates an increased risk of oral and pharyngeal cancer in individuals consuming the highest quantities of coffee; an OR lower than 1 indicates a decreased risk of oral and pharyngeal cancer in individuals consuming the highest quantities of coffee.

DerSimonianand Laird random-effects models were used to determine the overall estimates. The presence of publication bias was examined through the visual inspection of funnel plot asymmetry, and statistically tested using the Begg and Mazumdar rank correlation method (*p*<0.05 represents statistically significant publication bias). The estimation of possible missed studies was statistically tested with the Duval and Tweedie non-parametric “trim and fill” method.

## Results

- Search procedure and characteristics of the included studies

**Figure 1:**
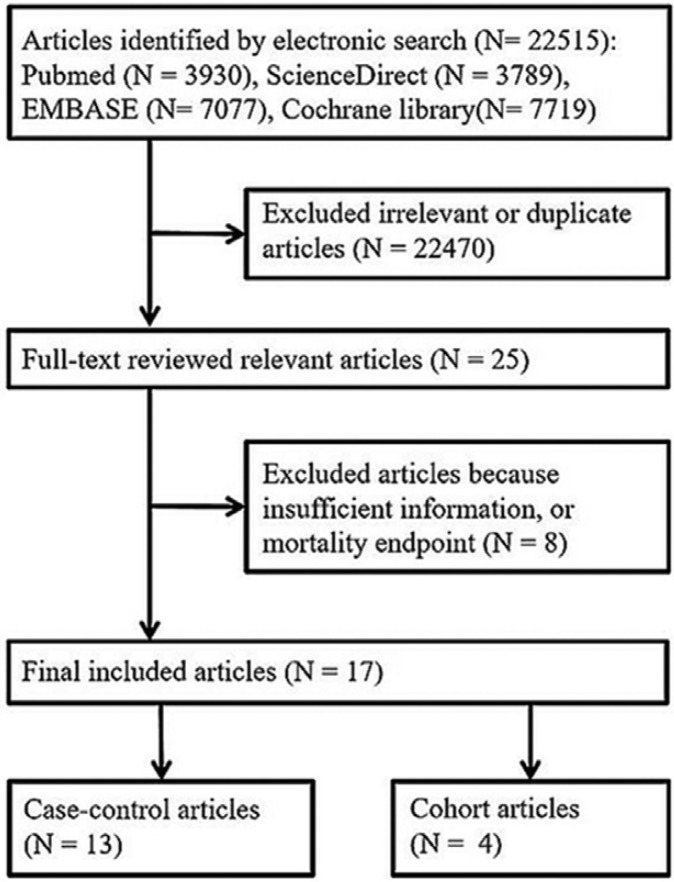
Flow chart of the article selection.

Figure 1 presents a flowchart that briefly overviews the search process for the trials included in the meta-analysis. Out of a total of 22,515 studies, we excluded 22,470 duplicates and irrelevant articles and filtered 25 for in-depth study and reading. After a thorough evaluation of the studies in question, we selected 17 studies with the desired data to proceed with the execution of the meta-analysis ( Table 1). This included assessing the quality of the studies, using the Newcastle-Ottawa Quality Scale (NOS) as previously described: patient population and selection, comparability of the study, and exposure. The quality score average was high with a final value of 6.06 with the 17 studies obtaining a score of 5 or more (ranging between 5 and 9) ( Table 2).

**Table 1:**
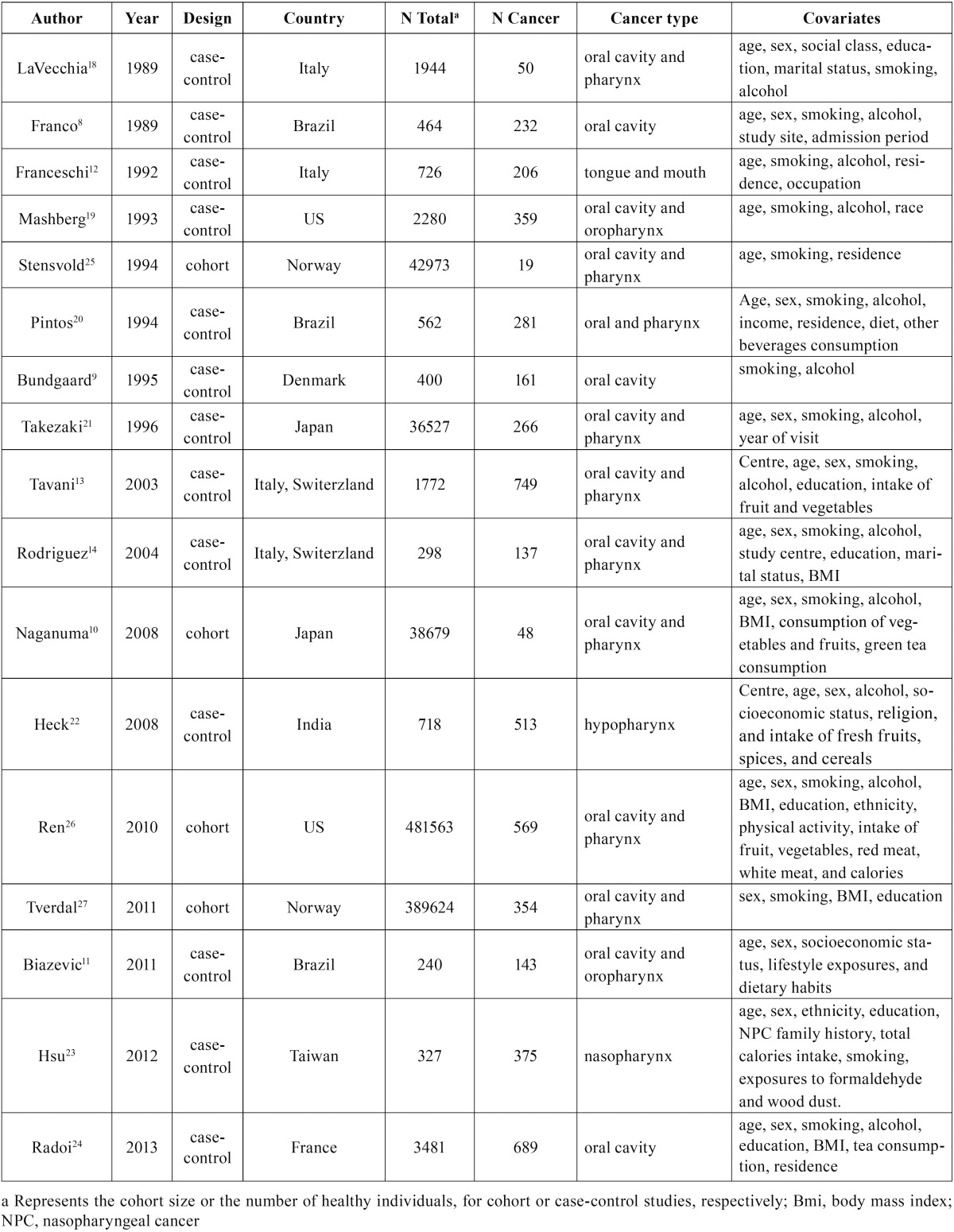
Data and characteristics of the included studies.

**Table 2:**
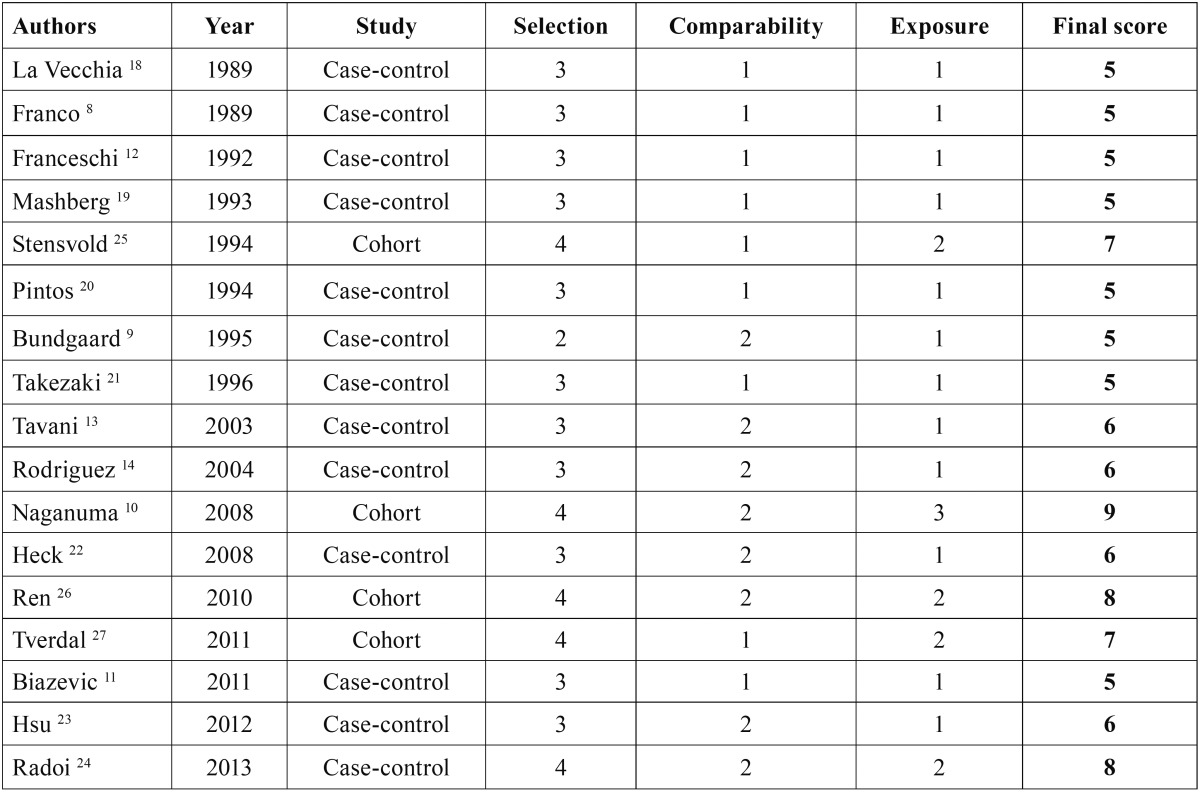
Quality of the selected studies by using the Newcastle–Ottawa scale.

Thirteen studies were case-control (8,9,11-14,18-24), whereas the remaining four were cohort studies (10,25-27). The majority of studies (n=8) were conducted in European countries, five were conducted in America (north or south) and four in Asia. All of the included studies provided adjusted measures (odds ratio, relative risk or hazard ratio) from multivariate models. Three of the included studies did not provide the corresponding confidence intervals. Thus, for these studies, standard errors were calculated based on the sample size of the groups and the 95% CI was further estimated.

- Quantitative Analysis

**Figure 2:**
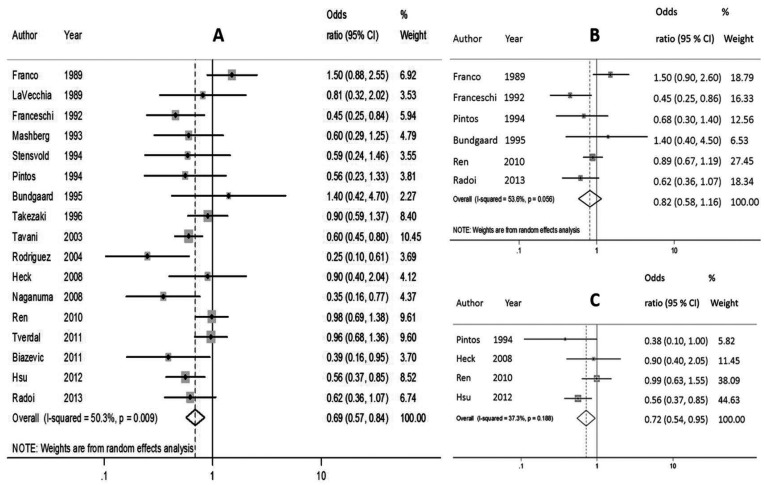
Forest plot of the meta-analysis regarding the relative risk of both oral and pharyngeal cancer (A), oral cavity cancer (B) and pharyngeal cancer (C) for highest coffee vs lowest coffee consumption using random effects analysis.

The results of the quantitative analysis revealed that coffee consumption is significantly associated with oral and pharyngeal cancer (z=3.63, *p*<.001), with the odds for cancer risk in individuals with the highest coffee consumptions are 1.45 times less than the odds for cancer risk in individuals with the lowest coffee consumptions (pooled OR = .69; 95% IC=.57-.84). The results are graphically represented in the forest plot (Fig. 2A). A significant (Χ2(16)=32.21, p=.009) and moderate (I2=50.3%) levels of heterogeneity was found across the studies ( Table 3).

**Table 3:**
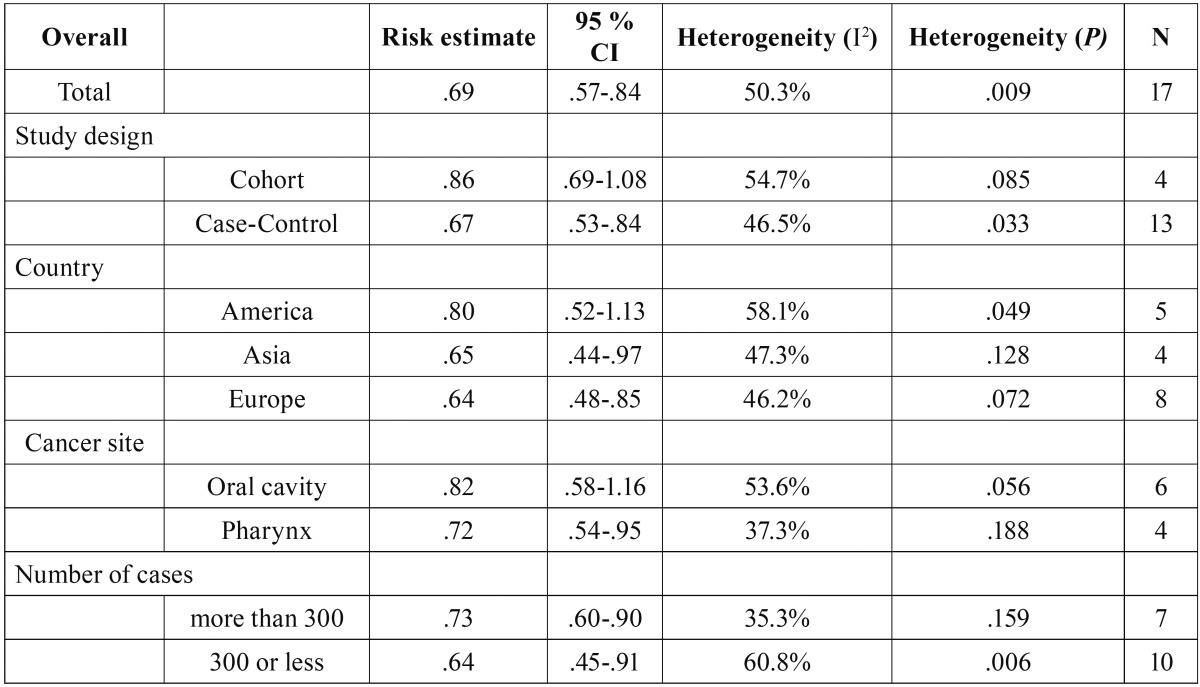
Summary of results of subgroup analyses.

In order to further explore the heterogeneity, subgroup analyses were conducted. When considering case-control studies alone, it was observed that the heterogeneity had a small decrease comparing with the overall analysis (I2=46.5%, Χ2 (12) =22.42, *p*=.033). Additionally, it was noted that the overall effect remains statistically significant (pooled OR = .67; 95% IC=.53-.84, z=3.48, *p*<.001). For the group of cohort studies, the level of heterogeneity was slightly higher (I2=54.7%) than the group of case-control studies, and non-significant (Χ2 (3) =6.62, *p*=.085). However, in contrast with the group of case-control studies, it was observed that the level of coffee consumption was not related with the presence/absence of oral/pharynx cancers (pooled OR = .86; 95% IC=.69-1.08; z=1.27, *p*=.202).

The conduction of meta-analyses stratified by the location of the country revealed a significant heterogeneity between studies conducted in the Americas (I2=58.1%; Χ2 (4) =9.56, *p*=.049). On the other hand, the level of heterogeneity was less pronounced in Asian (I2=47.3%; Χ2 (3) =5.69, *p*=.128) and European (I2=46.2%; Χ2 (7) =13.00, *p*=.072) countries. The summary of the other results of the subgroup analyses is presented in  Table 3.

With respect to the studies’ sample size, it was evident that studies with more than 300 cases had lower (I2=35.3%) and non-significant heterogeneity levels (Χ2 (6) =9.27, *p*=.159), whereas for studies with 300 or less cases, the heterogeneity between studies increased to moderate, and significant, levels (I2=60.8%; Χ2 (9) =22.96, *p*=.006).

In order to ascertain the impact of each study on the overall results, a sensitivity analyses were performed with a leave-one-out strategy (i.e. multiple meta-analyses were conducted, and in each, one of the studies was excluded individually). However, it was observed that the exclusion of a single study did not produce significant differences in overall effects when analysing the samples of oral and pharyngeal cancer together.

To explore a possible specific relationship of the coffee consumption with cancer location, in particular for oral cavity vs the pharynx, we performed a subgroup analysis regarding the oral cavity vs pharynx locations separately.

Among oral cavity cancer studies, the analysis with fixed effects revealed an absence of a statistically significant association between the consumption of higher levels of coffee and oral cancer risk (z=1.13, *p*=.257). The results are graphically represented in Figure 2B (pooled OR = .82; 95% IC=.58-1.16). A non-significant (Χ2(5)=10.77 *p*=.056) and moderate (I2=53.6%) heterogeneity was found across these studies.

The sensitivity analysis (leave-one-out) revealed that the exclusion of the study by Franco *et al.* (8) has a significant impact on the overall estimate. In particular, when this study is removed from the analysis, a significant association between coffee con-sumption and oral cavity cancer is observed (pooled OR = .77; 95% IC=.61-.95, z=2.40, *p*=.017). Furthermore, the level of hete-rogeneity among studies is reduced to a lower, non-significant level (Χ2(4)=5.46 *p*=.244, I2=26.7%), enabling the use of a fixed-effects model. Despite yielding a lower impact, it was also verified that the exclusion of the study of Bundgaard *et al.* (9) also contributes to an overall significant association.

Among the studies analysing pharyngeal cancers alone, we observed a significant association between coffee consumption and risk for cancer (z=2.34, *p*=.019). The results are graphically displayed on the forest plot (pooled OR = .72; 95% IC=.54-.95) (Fig. 2C). A non-significant, low/moderate heterogeneity was observed among the studies (Χ2(3)=4.79, *p*=.188, I2=37.3%).

In contrast with the results obtained on the oral cavity, the sensitivity analysis revealed that excluding a single study does not significantly affect the overall estimate on pharyngeal cancers.

- Publication bias

**Figure 3:**
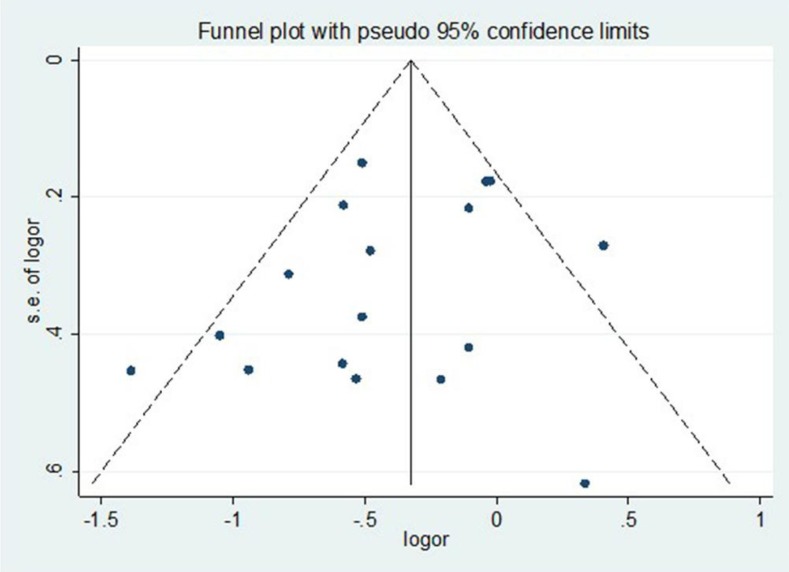
Begg’s funnel plot of asymmetry used to assess the publication bias for the included articles (vertical axis, standard error and horizontal axis, log hazard ratio).

Begg’s funnel plot was used to assess the publication bias for the included articles. The graphical representation suggests a re-duced asymmetry (Fig. 3). Begg’s test (z=.62, *p*=.537) and Egger’s test (t(16)=-.1.15, *p*=.267) revealed that there was no significant publication bias. Furthermore, using the “trim and fill” method, no missing studies were identified.

## Discussion

Beverages such as alcohol and mate drinking have been linked to an increased risk of oral and pharyngeal cancers (28). According to the results obtained in this meta-analysis, there is indeed a significant protective association between coffee consumption and the risk of oral and pharyngeal cancer. In fact, the observational, cohort, and case-control studies included in our analysis showed an inverse association between coffee consumption and oral/pharyngeal cancer, and in particular, we may infer that the likelihood of developing oral cancer in individuals who consume higher amounts of coffee is 1.45 times less than for individuals who consume small quantities of coffee or for non-consumers. Our results, including a larger number of studies and the robustness of the qualitative analysis confirm the results of previous systematic reviews and meta-analysis studies which have all suggested a protective effect of coffee drinking (15,16,29,30). Interestingly, we observed that this significant inverse association was clearer when we analysed studies on oral cancer containing cases from the pharynx or in pharyngeal cancers. The exact location of the cancers were sometimes ignored in studies or were under the designation of oral cancer (including cases from the pharynx and larynx). This is an important aspect that should be taken in to account in future works that are reported. An inverse association was also documented in other studies on cancers of liver, endometrial, and colorectal cancers (29).

The chemical and biological properties of coffee could explain in part the positive influence on oral and pharyngeal cancer risk reduction. Coffee beverages, additionally to caffeine, contains a variety of antioxidant and anti-mutagen agents including phenolic derivates (such as chlorogenic acid and polyphenol caffeic acid) and diterpenes (such as cafestol and kahweol), that could act as carcinogenic detoxifying agents on oral and pharyngeal mucosa (6). Further studies should determine the impact of coffee constituents as a chemopreventive agent against cancer especially oral and pharyngeal cancers. The concentration and dose of these agents should be determined along with the type of coffee beans (Robusta or Arabica), the method of roasting and the absence of collateral effects or others diseases. Taking into account of the popularity of coffee, this could be of particular interest. Whether high consumers of coffee have a lower intake of alcohol – a major risk factor for oral and pharyngeal cancers - is worth further investigation.

There are some limitations which have arisen during the execution of our meta-analysis. There was little information related to the type of coffee beans (Arabica or Robusta) or the brewing procedure, methodology used in the different studies we consulted, as well as the concentration of caffeine, and the size of the cups used. Moreover, there were different categories of coffee consumption in the studies, which did not allow for quantification of the association between oral cancer and the number of cups of coffee consumed. This meant that we only extracted the OR value to thereby compare the highest rate of consumption with the lowest rate. Another important aspect is the temperature of the coffee that almost in every study is not a controlled aspect. The temperature of oral beverages has been linked to some types of cancer (7,20). This should be an important aspect that should be controlled in future observational studies. On the other hand, we detected the presence of moderate (~50%) heterogeneity within the general analysis. This is a problem related with the nature and type of studies that are often reported in systematic reviews and meta-analyses (16). Nevertheless, when we proceed to subgroup analysis, heterogeneity was lower especially in analysis using studies from Europe, and in pharyngeal cancer locations. Additionally, the sample size used in studies including more than 300 cases of patients with oral and pharyngeal cancer showed a significantly lower heterogeneity value compared to studies which included fewer than 300 cases. Another possible limitation could be the existence of some covariates, such as tobacco and alcohol, two covariates that are mainly responsible for carcinogenic potential in the oral mucosa and may be not equitably distributed in the articles that were selected to our study.

Nevertheless, we think that our results are of interest and important for the comprehension of the role of coffee consumption on oral / pharyngeal cancers. First we have selected the case-control and cohort studies with a high quality score and large samples of cases and participants and performed an analysis reporting oral cavity and pharyngeal cancers but also analysing into a subgroup of oral cavity vs pharyngeal cancers. Secondly, we found no publication bias in our study.

We can conclude that the results obtained from this meta-analysis, confirm that there is an inverse association between high coffee consumption and the risk of oral/pharyngeal cancer, so we can infer that individuals who consume higher amounts of coffee are less likely to develop oral cancer. However, in the future, it may be necessary to determine the robustness of the results obtained in this study and to thus confirm the inverse association demonstrated, as well as establishing greater rigour and consistency between variables and co-variables, especially tobacco and alcohol.
